# Incidence of second primary malignancies in patients with thyroid cancer in the Turkish population

**DOI:** 10.3906/sag-1903-104

**Published:** 2019-10-24

**Authors:** Melia KARAKÖSE, İlker ÇORDAN, Mustafa CAN, Muhammet KOCABAŞ, Mustafa KULAKSIZOĞLU, Feridun KARAKURT

**Affiliations:** 1 Division of Endocrinology and Metabolism, Department of Internal Medicine,Faculty of Medicine, Necmettin Erbakan University, Konya Turkey

**Keywords:** Thyroid cancer, second primary cancer, breast cancer

## Abstract

**Background/aim:**

Thyroid cancer is the most common endocrine malignancy. Recently the incidence has been increasing faster compared to other malignancies. Different studies have shown that the incidence of breast cancer in patients followed due to thyroid cancer has increased, and vice versa. The aim of this study was to evaluate the frequency of second primary cancers in the follow-up of patients with thyroid cancer.

**Materials and methods:**

In this study, 1196 patients with thyroid cancer were evaluated in the Necmettin Erbakan University Meram Medical School’s Department of Endocrinology between 2004 and 2018. Demographic characteristics and radiological and pathological results of the patients were recorded. The presence of accompanying second malignancies in patients with thyroid cancer was investigated.

**Results:**

In our study, 985 (82.4%) women (mean age: 46.1 ± 13.3 years) and 211 (17.6%) men (mean age: 49.9 ± 14.2 years) were evaluated. The median follow-up was 63 months (2–164 months). Of the 1196 patients, 1126 (94.1%) had no additional cancer and 70 (5.9%) patients had a second malignancy. The accompanying second malignancies were breast cancer in 24 (2%) patients, skin cancer in 8 (0.7%) patients, renal cell cancer in 5 (0.4%) patients, lung cancer in 5 (0.4%) patients, colon cancer in 5 (0.4%) patients, lymphoma in 5 (0.4%) patients, endometrial cancer in 4 (0.3%) patients, and 14 cases of other rare types of cancer.

**Conclusion:**

In our study, it was found that the most common second primary malignancy in patients with thyroid cancer was breast cancer. However, other cancers (skin cancer, renal cell cancer, lymphoma, and colon, lung, or endometrial cancer) may occur in patients with thyroid cancer.

## 1. Introduction

Thyroid cancer is the most common endocrine malignancy. The incidence continues to increase worldwide [1]. Thyroid cancer accounts for approximately 1.0%–1.5% of all new cancer cases [2]. It is most common in women between the ages of 30 and 70 and is 2.9 times more likely to occur in women than in men. Although the incidence of thyroid cancer is increasing, mortality due to thyroid cancer is decreasing. This is because of early diagnosis and effective treatment with surgery and radioactive iodine (RAI) treatment [3]. More than 90% of thyroid cancers comprise differentiated thyroid cancers, including papillary and follicular carcinoma types [4]. Less common types of thyroid cancers are medullary thyroid cancer (5%–9%) and anaplastic thyroid cancer (1%–2%) [5].

Increased incidence of thyroid cancer and good prognosis has raised concerns about the development of second primary cancers, especially after RAI treatment. In addition, thyroid cancers may also occur as secondary neoplasms after other cancers. Different studies have shown that the incidence of breast cancer in patients followed due to thyroid cancer has increased and also the risk of thyroid cancer has increased in the long-term follow-up of breast cancer patients. It has also been found that the risk of other second primary cancers, such as prostate, kidney, and adrenal cancer, increases in thyroid cancer patients [6–8]. The aim of this study was to evaluate the frequency of second primary cancers in the follow-up of patients with thyroid cancer.

## 2. Materials and methods

A total of 1196 patients who were followed with the diagnosis of thyroid cancer between 2004 and 2018 were included in the retrospective study. The Necmettin Erbakan University Meram Medical School Ethics Committee approved the study with approval no. 2018/1647 dated 04.01.2019. The demographic, clinical, radiological, and pathological data of the patients were recorded from the patient files. No patients had a history of thyroid or neck surgery for nonthyroidal cancer or neck irradiation. Papillary thyroid carcinomas measuring 10 mm or less in diameter were classified as papillary thyroid microcarcinoma. The presence of a second accompanying malignancy of patients was investigated.

### 2.1. Statistical analysis

Statistical analysis was performed using SPSS 22.0 (IBM Corp., Armonk, NY, USA). Continuous variables were given as mean ± standard deviation if the distribution was normal, and as median (minimum–maximum) if the distribution was not normal. In the comparison of independent groups’ differences, the significance test of the difference between the two means (independent samples t-test) was used when the parametric test assumptions were met. The Mann–Whitney U test was used to compare independent groups’ differences when the parametric test assumptions were not provided. For differences, P < 0.05 was considered statistically significant.

## 3. Results

**Figure 1:**
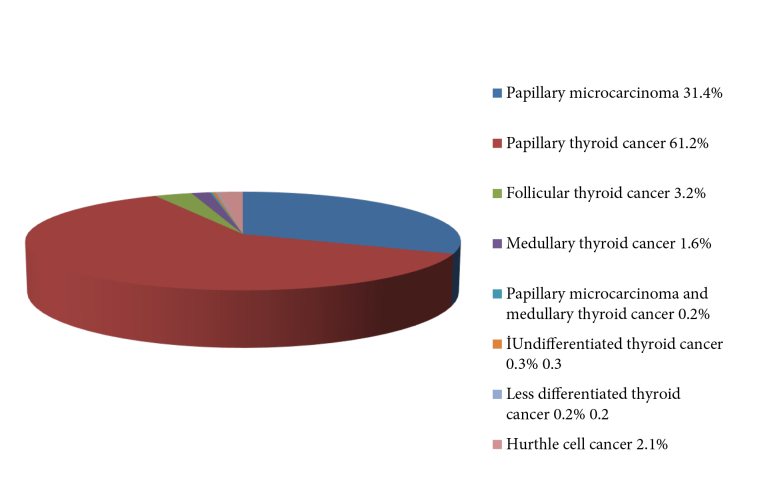
Thyroid cancer pathological subtypes of all patients.

The study population included 1196 patients, 985 (82.4%) of whom were female and 211 (17.6%) of whom were male. The mean age of women was 46.1 ± 13.3 years and the mean age of men was 49.9 ± 14.2 years. The median follow-up was 63 months (2–164 months). The pathological diagnoses were papillary microcarcinoma in 375 (31.4%) patients, papillary thyroid cancer in 732 patients (61.2%), follicular thyroid cancer in 38 (3.2%) patients, medullary thyroid cancer in 19 (1.6%) patients, papillary microcarcinoma and medullary thyroid cancer in 2 (0.2%) patients, undifferentiated thyroid cancer in 3 (0.3%) patients, less differentiated thyroid cancer in 2 (0.2%) patients, and Hurthle cell cancer in 25 (2.1%) patients (Figure 1). Of the 1196 patients, 1126 (94.1%) had no additional cancer and 70 (5.9%) had a second primary malignancy.

**Figure 2:**
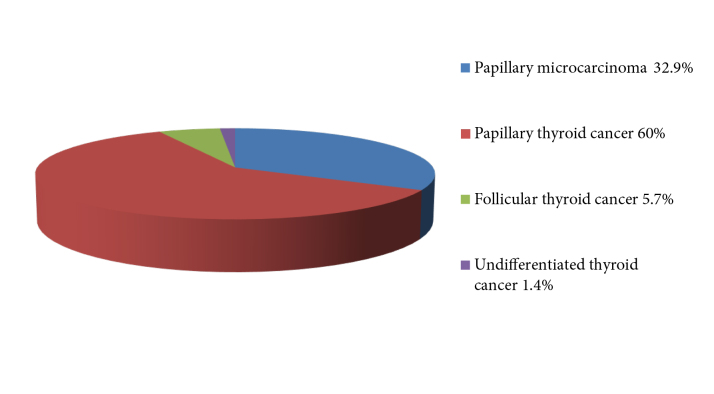
Thyroid cancer pathological subtypes of patients in whom second primary malignancy was detected

Of the 70 patients with a second primary malignancy, 55 (78.6%) were female and 15 (21.4%) were male. The mean age of female patients was 52.1 ± 10.6 years and the mean age of male patients was 61.8 ± 11.6 years. The mean age of the male patients was significantly higher than that of the female patients (P = 0.003). The mean follow-up period for thyroid cancer was 61.8 ± 39.2 months and the mean follow-up period for the second primary malignancy was 69.5 ± 48.9 months. Of these 70 patients, 23 (32.9%) had papillary microcarcinoma, 42 (60%) had papillary thyroid cancer, 4 (5.7%) had follicular thyroid cancer, and 1 (1.4%) had undifferentiated thyroid cancer (Figure 2). The accompanying second malignancies were breast cancer in 24 (2%) patients, skin cancer in 8 (0.7%) patients, renal cell cancer in 5 (0.4%) patients, lung cancer in 5 (0.4%) patients, colon cancer in 5 (0.4%) patients, lymphoma in 5 (0.4%) patients, endometrial cancer in 4 (0.3%) patients, and other rare cancer types in 14 patients (Table).

**Table:** Second primary cancers in the follow-up of patients with thyroid cancer.

Second primary cancer	n (%)
Breast cancer	24 (2%)
Skin cancer	8 (0.7%)
Lung cancer	5 (0.4%)
Colon cancer	5 (0.4%)
Renal cell cancer	5 (0.4%)
Lymphoma	5 (0.4%)
Endometrial cancer	4 (0.3%)
Prostate cancer	3 (0.3%)
Ovarian cancer	3 (0.3%)
Carcinoid tumor	1 (0.1%)
Parotid cancer	1 (0.1%)
Bladder cancer	1 (0.1%)
Kaposi’s sarcoma	1 (0.1%)
Gastric cancer	1 (0.1%)
Hairy cell leukemia	1 (0.1%)
Nasopharyngeal cancer	1 (0.1%)
Central nervous system cancer	1 (0.1%)

Of the 1196 patients included in our study, 855 (71.5%) received RAI treatment and 341 (28.5%) did not. The median dose of RAI treatment was 150 mCi (min–max: 100–800) and mean RAI treatment dose was 146 ± 73 mCi. Of the 70 patients with second primary malignancies, 32 were diagnosed with thyroid cancer first and then diagnosed with the second primary malignancy. Of these 32 patients, 25 received RAI treatment. The median RAI dose of patients who received RAI was 150 mCi (min–max: 100–650); mean RAI dose was 181 ± 144 mCi. These patients were diagnosed with a second primary malignancy after a mean of 39 ± 33 months after thyroid cancer diagnosis (median: 38 months; min–max: 1–118 months). Of the 70 patients with a second primary malignancy, 38 were diagnosed with the malignancy prior to thyroid cancer and 25 of these patients had RAI. The median RAI dose of patients who received RAI was 150 mCi (min–max: 100–400); mean RAI dose was 160 ± 68 mCi. There was no difference between patients with another malignancy prior to thyroid cancer diagnosis and those diagnosed with a second primary malignancy after thyroid cancer diagnosis in terms of RAI dose (P= 0.473).

## 4. Discussion

Thyroid cancer is the most common endocrine malignancy. Recently, the incidence of thyroid cancer has increased faster than that of other cancers. Despite the increased incidence of thyroid cancer, early diagnosis and effective use of treatment modalities such as surgery and RAI treatment have decreased mortality [3]. Increased incidence of thyroid cancer and good prognosis have raised concerns about the development of second primary malignancies, especially after RAI treatment. The reasons for the increased risk of second primary malignancies in patients with thyroid cancer are not fully known. Possible causes include RAI treatment, age, genetic predisposition, hormonal changes, and smoking [9–11].

In a study of 2031 patients with differentiated thyroid cancer, Silva et al. found that the mean age of thyroid cancer patients was 48 years, 83% were female, and 17% were male [12]. In our study 1196 patients were included, 82.4% of whom were female and 17.6% of whom were male. The mean age of women was 46.1 ± 13.3 years and the mean age of men was 49.9 ± 14.2 years. 

In the study of Izkhakov et al., 1032 patients with thyroid cancer were included and 85.4% of the patients had papillary thyroid cancer, 9.2% had follicular thyroid cancer, 3.4% had medullary thyroid cancer, and 1.9% had less differentiated thyroid cancer. In their study, a second primary malignancy was found in 8.9% of patients. In their study, breast, brain, urinary system, and lung cancers in women and urinary system and prostate cancers in men were found to be the accompanying cancer types [13]. In the study of Silva-Vieira et al., a second malignancy was detected in 6.4% of 2031 differentiated thyroid cancer patients. Forty (1.9%) patients had breast, 24 (1.1%) had genitourinary, 23 (1.1%) had gastrointestinal, 12 (0.5%) had hematologic, and 12 (0.5%) had lung-pleural cancer [9]. In our study, in terms of thyroid cancer subtypes, 92.6% of patients had papillary thyroid cancer, 3.2% had follicular thyroid cancer, 1.6% had medullary thyroid cancer, 0.2% had papillary microcarcinoma and medullary thyroid cancer, 0.3% had undifferentiated thyroid cancer, 0.2% had less differentiated thyroid cancer, and 2.1% had Hurthle cell thyroid cancer. In our study, the rate of second primary malignancies in thyroid cancer patients was 5.9%. We found breast cancer in 24 (2%) patients, skin cancer in 8 (0.7%) patients, renal cell cancer in 5 (0.4%) patients, lung cancer in 5 (0.4%) patients, colon cancer in 5 (0.4%) patients, lymphoma in 4 (0.3%) patients, endometrial cancer in 4 (0.3%) patients, and other rare cancers in 14 patients.

In their study, Sandeep et al. showed that there was a second primary malignancy in 2821 of 39,002 patients with thyroid cancer. When thyroid cancer patients with accompanying second malignancies were examined in terms of subtypes of thyroid cancer, they found that 39.8% had papillary thyroid cancer, 13.5% had follicular thyroid cancer, and 3% had medullary thyroid cancer [7]. In our study, of the 70 patients with thyroid cancer who had a second primary malignancy, 23 (32.9%) had papillary microcarcinoma, 42 (60%) had papillary thyroid cancer, 4 (5.7%) had follicular thyroid cancer, and 1 (1.4%) had undifferentiated thyroid cancer. In the metaanalysis of Nielsen et al., the risk of thyroid cancer was increased as a second cancer following breast cancer (OR = 1.55, 95% CI: 1.44–1.67). In the same metaanalysis, the risk of breast cancer was also increased after thyroid cancer (OR = 1.32, 95% CI: 1.23–1.42) [14].

In 2018, the incidence of all cancers in Turkey was detected as 225.1 per 100,000 persons (0.2%) [15]. In our study, a second primary malignancy was detected in 70 (5.9%) of 1196 patients with thyroid cancer. In our study, the rate of second primary malignancy in patients with thyroid cancer was detected to be higher than in the general population. In our study, rates of breast, lung, and colon cancers were found to be higher in patients with thyroid cancer than in the general population. The incidence of breast cancer in Turkey is 46.8 per 100,000 persons. In the study of Silva et al., breast cancer was detected in 1.9% of 2031 differentiated thyroid cancer patients [12]. In our study, breast cancer was detected in 2% of 1196 patients with thyroid cancer. The incidence of lung cancer in Turkey is 53.8 per 100,000 males and 8.2 per 100,000 females. In the study of Silva et al., lung/pleural cancer was detected in 0.5% of 2031 differentiated thyroid cancer patients [12]. In our study, lung cancer was detected in 0.4% of 1196 patients with thyroid cancer. The incidence of colon cancer in Turkey is 21.8 per 100,000 males and 13.2 per 100,000 females. In the study of Silva et al., gastrointestinal tract cancer was detected in 1.1% of 2031 differentiated thyroid cancer patients [12]. In our study, colon cancer was detected in 0.4% of 1196 patients with thyroid cancer.

In a study conducted by Lang et al. with 895 thyroid cancer patients, 643 (71.8%) of the patients received RAI treatment. After a median follow-up of 93.5 months (range: 23.4–570.8), 64 (7.2%) patients had developed a second primary malignancy [14]. The risk of second primary malignancy was significantly higher in the group treated with RAI treatment than in the group without RAI treatment. In their study, 80–240 mCi cumulative RAI treatment dose was identified as the only independent risk factor for second primary malignancies [14]. Of the 1196 patients included in our study, 855 (71.5%) received RAI treatment and 341 (28.5%) did not. The median dose of RAI treatment was 150 mCi (min–max: 100–800) and mean RAI treatment dose was 146 ± 73 mCi. Of the 70 patients with a second primary malignancy, 32 were diagnosed with thyroid cancer first and then diagnosed with the second primary malignancy. Of these 32 patients, 25 received RAI treatment. The median RAI dose of patients who received RAI was 150 mCi (min–max: 100–650); mean RAI dose was 181 ± 144 mCi. These patients were diagnosed with a second primary malignancy after a median of 39 ± 33 months after thyroid cancer diagnosis (median: 38 months, min–max: 1–118 months). Of the 70 patients with second primary malignancy, 38 were diagnosed with malignancy prior to thyroid cancer and 25 of these patients had RAI. The median RAI dose of patients who received RAI was 150 mCi (min–max 100–400); mean RAI dose was 160 ± 68 mCi. There was no difference between patients with another malignancy prior to thyroid cancer diagnosis and those diagnosed with a second primary malignancy after thyroid cancer diagnosis in terms of RAI dose (P = 0.473).

In conclusion, it was found that the most common second primary malignancy in patients with thyroid cancer was breast cancer, and many other cancers (skin cancer, renal cell cancer, lymphoma, colon, lung, and endometrial cancer) were also detected as second primary cancer in patients with thyroid cancer. There is a need for further studies with more cases and longer follow-up.
